# Assessing the Efficacy of AI Segmentation in Diagnostics of Nine Supernumerary Teeth in a Pediatric Patient

**DOI:** 10.3390/diagnostics13233563

**Published:** 2023-11-29

**Authors:** Rasa Mladenovic, Zoran Arsic, Stefan Velickovic, Milan Paunovic

**Affiliations:** 1 Department for Dentistry, Faculty of Medical Sciences, University of Kragujevac, 34000 Kragujevac, Serbia; velickovicstefan91@gmail.com; 2 Department for Dentistry, Faculty of Medicine, University of Pristina, 38220 Kosovska Mitrovica, Serbia; 3 Department of Surgery, Faculty of Medical Sciences, University of Kragujevac, 34000 Kragujevac, Serbia; mpaunovic1969@gmail.com

**Keywords:** artificial intelligence, 3D segmentation, supernumerary teeth

## Abstract

We present a very rare case of a child with nine supernumerary teeth to analyze the potential, benefits, and limitations of artificial intelligence, as well as two commercial tools for tooth segmentation. Artificial intelligence (AI) is increasingly finding applications in dentistry today, particularly in radiography. Special attention is given to models based on convolutional neural networks (CNN) and their application in automatic segmentation of the oral cavity and tooth structures. The integration of AI is gaining increasing attention, and the automation of the detection and localization of supernumerary teeth can accelerate the treatment planning process. Despite advancements in 3D segmentation techniques, relying on trained professionals remains crucial. Therefore, human expertise should remain key, and AI should be seen as a support rather than a replacement. Generally, a comprehensive tool that can satisfy all clinical needs in terms of supernumerary teeth and their segmentation is not yet available, so it is necessary to incorporate multiple tools into practice.

Supernumerary teeth, also referred to as extra teeth, represent dental irregularities in which there are additional teeth present in the oral cavity beyond the usual dentition [1]. Non-syndromic supernumerary teeth, on the other hand, are extra teeth that develop independently of specific genetic syndromes. These extra teeth emerge as anomalies during dental development and do not accompany any broader medical conditions. They can manifest individually or in multiple occurrences, with their appearance being influenced by a combination of genetic and environmental factors. Non-syndromic supernumerary teeth are typically located in various positions within the mouth, but they are commonly observed between the central maxillary incisors [2]. Detecting the presence of supernumerary teeth often involves observing wider gaps or misalignment of erupted teeth, and a definitive diagnosis relies on radiographic evaluation [3]. In complex cases where teeth are interposed, multiple retroalveolar X-rays may be required in order to precisely determine the exact position of the supernumerary tooth and its relationship with the permanent tooth germ. While conventional X-rays provide initial insights, advanced imaging techniques like CBCT offer a more accurate perspective of the tooth’s location and its interaction with the surrounding structures [3,4,5].

The clinical significance of diagnosing supernumerary teeth is extremely important for several reasons. In addition to contributing to the preservation of oral health, the diagnosis plays a crucial role in achieving the desired aesthetic appearance of the smile and face [1]. Oral and jaw functionality is also improved while simultaneously preventing complications. Furthermore, the emotional and social aspects are not to be overlooked, as individuals with supernumerary teeth often face challenges in terms of self-confidence and social interaction. Therefore, the timely diagnosis of supernumerary teeth has broader implications for oral health, aesthetics, functionality, and the psychosocial well-being of patients. Artificial intelligence (AI) has made significant advancements in the field of 3D segmentation for dental radiology [6,7]. AI techniques, such as machine learning and deep learning, have shown promising results in automating the segmentation process, improving efficiency, and reducing the dependency on manual labor. There are two primary methods of segmentation: manual and automated. In manual segmentation, a trained operator or clinician uses specialized software tools to manually trace or outline the desired structures on each 2D slice of the cone beam computed tomography (CBCT) volume. These outlines are then connected to create a 3D model. Automated segmentation utilizes computer algorithms and techniques to automatically identify and segment the desired structures [8]. These algorithms can be based on various approaches, such as thresholding, region growing, edge detection, machine learning, or deep learning techniques. Automated segmentation can be faster and more consistent than manual segmentation, but it may require training data or optimization to achieve accurate results.

Our aim was to present an exceptionally rare case of a patient with nine supernumerary teeth (Figure 1), as well as to showcase the performance and capabilities of two commercial AI tools (Figure 2 and Figure 3) for automatic recognition and 3D segmentation in dental radiology (Table 1).

**Figure 1:**
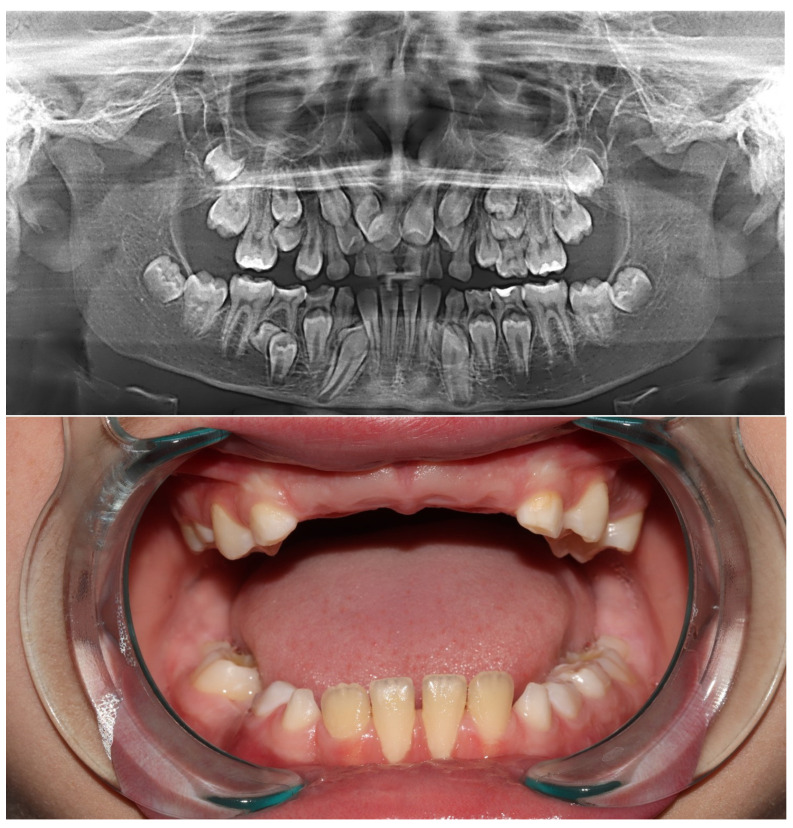
The patient was admitted to the Dental Clinic of the Faculty of Medical Sciences, University of Kragujevac due to unerupting teeth in the frontal region. His parents reported an unremarkable medical history. During the general physical examination of the hands and the phenomenon of shoulders close to the front of the body, it did not indicate that it was Cleidocranial Dysplasia (CCD). However, a molecular genetic analysis was proposed to exclude a diagnosis of CCD. Considering the patient’s age (eleven years), a retroalveolar X-ray was initially performed, followed by a panoramic X-ray. After a thorough examination of the panoramic X-ray, primary teeth were extracted in the frontal region, and it was decided that a CBCT scan would be performed to obtain more data regarding the supernumerary teeth.

**Figure 2:**
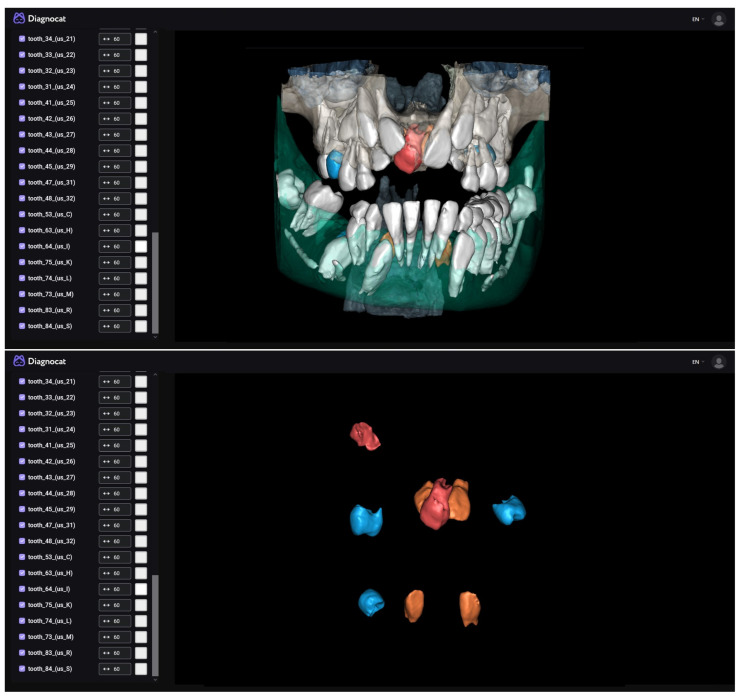
The Diagnocat Inc. (San Francisco, CA, USA) AI system exploits a set of pre-trained semantic segmentation networks based on internally modified, fully convolutional 3D U-Net architecture to obtain voxel-perfect segmentation. Image analysis takes only a few minutes (around 5 min for CBCT), and the software generates a detailed report on the condition of each tooth, along with suggestions for further diagnosis (DICOM format is required). In just a few steps, segmentation is achieved in the integrated 3D viewer. Alongside the simple selection of desired structures and teeth, this tool offers the option of STL export, which is of exceptional importance for digital dentistry. Exported 3D models can be valuable for learning as well as for creating guides for potential surgical intervention. In our case, this tool recognized all the supernumerary teeth through automatic segmentation. A mesiodens is shown, all lateral incisors are duplicated, three duplicated premolars and one distomolar are present in each jaw. Diagnocat is a fully automated tool, so it does not allow for additional settings in analysis or segmentation, only the export of the 3D model. By simply selecting the structures, a simulation of the removal of supernumerary teeth can be prepared.

**Figure 3:**
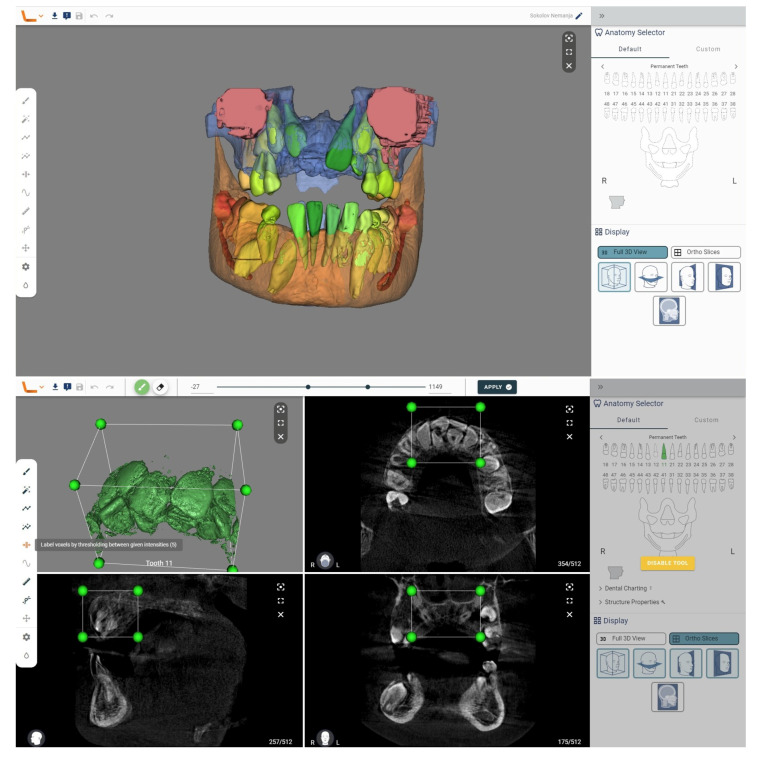
We analyzed a CBCT scan of the same patient using another tool, the Virtual Patient Creator (Relu, Leuven, Belgium). The tool did not recognize supernumerary teeth in the automatic analysis. But this tool offers numerous additional capabilities that can aid in diagnosing supernumerary teeth. After the initial segmentation, postprocessing steps can be applied to refine the segmented structures. By selecting the structure, in 2D cross-sections, a region can be identified for further analysis using the section labeled voxels by thresholding between given the intensities. This may involve removing outliers, smoothing the surface, filling gaps, or performing other operations to improve the accuracy and quality of the segmentation results. Once the segmentation is complete, the segmented structures can be visualized in 3D and further analyzed.

Tools based on artificial intelligence are increasingly finding applications in dentistry, and a recently published systematic review highlights numerous benefits that this technology offers [9]. These algorithms hold promise in visual diagnostics of dental issues, potentially reducing the workloads of oral health professionals and making diagnosis and treatment more accessible in developing countries.

The utilization of artificial intelligence (AI) in the diagnosis of supernumerary teeth is an emerging field with potential applications. AI algorithms can analyze dental radiographic images, such as panoramic radiographs [10] or CBCT scans, to automatically detect and localize supernumerary teeth [5]. Image-processing algorithms automatically recognize any abnormalities, significantly expediting the diagnostic process. Based on data obtained through X-ray diagnostics, AI enables precise treatment planning for the removal of supernumerary teeth. Moreover, algorithms can suggest optimal strategies for tooth extraction or repositioning to achieve the desired aesthetic and functional outcomes. The potential of AI as an invaluable tool for patient and parent education is also of paramount importance. Through simulations and animations, patients can visually and interactively gain a better understanding of the treatment plan, facilitating their informed decision making. What is particularly important in the treatment of supernumerary teeth are the time constraints of the operation, as these cases mostly involve younger patients; with rapid diagnosis and surgical simulation, we can save time in the process. In order to preserve tissue, STL export is of paramount importance, as it allows us to create a 3D guide as a foundation for minimally invasive surgical intervention [5].

It is important to note that the utilization of AI in the diagnostics of supernumerary teeth is still an evolving field, and the development of accurate and reliable AI models requires access to large, diverse, and well-annotated datasets. Additionally, human expertise and clinical judgment remain crucial in the interpretation of results provided by AI systems. AI should be used as a supportive tool to aid dental professionals in their decision-making processes rather than as a substitute for professional expertise. It is important to note that while 3D segmentation techniques have advanced significantly, they still rely on the expertise of trained professionals to ensure accurate results and clinical interpretations. In general, all available AI tools in dental radiology are still in development, and it is not possible to single out one comprehensive tool that can fully meet the needs of clinicians. Therefore, it is necessary to combine multiple tools to harness the potential of AI.

## Figures and Tables

**Table 1:** Main features of AI segmentation tools.

Features	Diagnocat (San Francisco, CA, USA)	Relu (Leuven, Belgium)
Format for analysis	DICOM	DICOM
Segmentation duration	Few minutes	Few minutes
Support STL export	Yes	Yes
Integrated 3D viewer	Yes	Yes
Recognizing supernumerary teeth	Yes	No
Additional settings for segmentation	No	Yes

## Data Availability

All relevant data are within the manuscript.
